# **Count data models for outpatient health services utilisation**

**DOI:** 10.1186/s12874-022-01733-3

**Published:** 2022-10-05

**Authors:** Nurul Salwana Abu Bakar, Jabrullah Ab Hamid, Mohd Shaiful Jefri Mohd Nor Sham, Mohd Nor Sham, Anis Syakira Jailani

**Affiliations:** 1 Centre for Health Policy Research, Institute for Health Systems Research, National Institutes of Health, Ministry of Health, Shah Alam, Malaysia; 2 Centre for Health Equity Research, Institute for Health Systems Research, National Institutes of Health, Ministry of Health, Shah Alam, Malaysia; 3 Centre for Health Economics Research, Institute for Health Systems Research, National Institutes of Health, Ministry of Health, Shah Alam, Malaysia; 4 Centre for Health Outcome Research, Institute for Health Systems Research, National Institutes of Health, Ministry of Health, Shah Alam, Malaysia

**Keywords:** Healthcare utilisation, Outpatient, Count model, Zero-inflated model, Health behavioral model

## Abstract

**Background:**

Count data from the national survey captures healthcare utilisation within a specific reference period, resulting in excess zeros and skewed positive tails. Often, it is modelled using count data models. This study aims to identify the best-fitting model for outpatient healthcare utilisation using data from the Malaysian National Health and Morbidity Survey 2019 (NHMS 2019) and utilisation factors among adults in Malaysia.

**Methods:**

The frequency of outpatient visits is the dependent variable, and instrumental variable selection is based on Andersen’s model. Six different models were used: ordinary least squares (OLS), Poisson regression, negative binomial regression (NB), inflated models: zero-inflated Poisson, marginalized-zero-inflated negative binomial (MZINB), and hurdle model. Identification of the best-fitting model was based on model selection criteria, goodness-of-fit and statistical test of the factors associated with outpatient visits.

**Results:**

The frequency of zero was 90%. Of the sample, 8.35% of adults utilized healthcare services only once, and 1.04% utilized them twice. The mean-variance value varied between 0.14 and 0.39. Across six models, the zero-inflated model (ZIM) possesses the smallest log-likelihood, Akaike information criterion, Bayesian information criterion, and a positive Vuong corrected value. Fourteen instrumental variables, five predisposing factors, six enablers, and three need factors were identified. Data overdispersion is characterized by excess zeros, a large mean to variance value, and skewed positive tails. We assumed frequency and true zeros throughout the study reference period. ZIM is the best-fitting model based on the model selection criteria, smallest Root Mean Square Error (RMSE) and higher R2. Both Vuong corrected and uncorrected values with different Stata commands yielded positive values with small differences.

**Conclusion:**

State as a place of residence, ethnicity, household income quintile, and health needs were significantly associated with healthcare utilisation. Our findings suggest using ZIM over traditional OLS. This study encourages the use of this count data model as it has a better fit, is easy to interpret, and has appropriate assumptions based on the survey methodology.

**Supplementary Information:**

The online version contains supplementary material available at 10.1186/s12874-022-01733-3.

## Background

Count data arising from measuring health care utilisation is a common outcome in health research, especially from national survey data. Count refers to the number of times an event occurs [1] and usually exhibits a skewed distribution. Often times, the presence of excess zeros and long positive tails leads to data dispersion. With this distinct characteristic, the assumption of normality is violated and a need for probability distribution approaches to handle the dispersions [2].

Count-valued outcomes are typically modeled using discrete distributions, such as the Poisson or negative binomial distributions [3]. In such cases, the data could either be unmodified, zero-inflated or zero-truncated relative to the standard model (linear or logistic regression model), where flexible mixture distributions are often needed to accommodate the unique features of the data. Previous studies have compared the overall performance of count regression models (Poisson, negative binomial, and their zero-inflated and hurdle variants) in modeling an outcome variable with extra zeros [3],[4]. Marginalized zero-inflated model also commonly used in exploring health care data[5, 6]. Other types of model such as Waring regression allows to distinguish between unobserved heterogeneity [7], hyper-Poisson regression utilizes the mean of the regressors [8] as well as Generalized Poisson model caters for under-dispersion property [9] were explored but the characteristics of these model not of interest in this current study.

Several estimation approaches have been developed to address zero-modified count data such as the application of the zero-inflated model in the discipline of arts [10, 11] and two-part or hurdle models in healthcare [12, 13]. While these models vary in terms of their distributional assumptions and parametric forms, they incorporate an underlying two-part model: a logistic part for excess zeros and a count part catering for zero and non-zero observations [5, 6].

Healthcare utilisation refers to being in contact with a certified medical or health facility, involving the process of seeking care to prevent or treat health problems [14]. The utilisation of healthcare services is the result of a complex decision-making process with multiple determinants, often occurring from the interaction between contextual and individual factors. These include individual characteristics, access to health services, and organization of the healthcare system [15]. Although utilisation of healthcare services is primarily decided by the choices of patients, the factors leading to such decisions are not merely individual preferences, but more complex choices involving the institutional, socioeconomic, and cultural backgrounds of the individual.

The Andersen Healthcare Utilisation Model (Andersen’s Behavioral Model of Health Services Use) is one of the most popular models of healthcare utilisation [16, 17]. Andersen’s model focuses on the social and economic factors that determine the use of health care. This model explains that healthcare utilisation depends on various factors ranging from the propensity of individuals to use services, the ability of individuals to access services, and individual’s health condition, each of which is represented by predisposing, enabling, and need factors. Predisposing characteristics are demographic variables that make some individuals more likely to use healthcare services than others. Enabling factors measure individuals’ ability to access health care from an economic standpoint. Need variables include risk factors for diseases, individual health states, and experiences of diseases that lead to seeking medical assistance. Need factors are the strongest predictors of healthcare utilisation, followed by enabling and predisposing factors [18, 19]. This study uses variables based on the Andersen Behavioral Model to identify associated factors in outpatient health care utilisation in Malaysia.

This study aims to identify the best-fitting count model for outpatient health care utilisation using data from the Malaysian National Health and Morbidity Survey 2019. We estimated different models and used several model selection criteria to identify the best-fitting criteria. This study also identifies the factors of health care utilisation among adults in Malaysia, which are vital for healthcare planners and managers.

## Methods

### Data source

This study utilized data from the National Health and Morbidity Survey (NHMS) 2019, a cross-sectional household survey in Malaysia conducted every four years to gather community-based data for health care utilisation and needs. The NHMS uses a two-stage stratified cluster sampling method conducted through face-to-face interviews. Details of the survey method are described in the official report [20]. The survey captures details on socio-demographic, health status, health problems, household income, and utilisation patterns, including frequency, service provider, and payment sources. Adults aged 18 years and older were included in this study. This study captured outpatient visits in the last 14 days for both public and private facilities. From a total of 11,674 sampled populations, only 8.1% reported utilized outpatient care at least once. The short reference period led to two types of zero: true zero reflects non-users because they did not get sick during the reference period, while frequency zero reflects individuals who fell sick during the reference period but did not seek care.

### Theoretical approaches and studied variables

This study utilized Andersen’s health behavioral model [16] to determine the predisposing (demographic), enabling (personal/family), and predictive (perceived/evaluated) factors of seeking outpatient care with the availability of the best presented variables collected from the NHMS The independent variables were selected based on the Andersen model. The dependent variable was the frequency of outpatient visits, and the selection of instrumental variables was done accordingly. Total household income was log-transformed using Ln(X), and imputation was performed on missing values based on working status and education level of the same group stratification. Statistical analysis was performed using STATA 14 (Stata Corporation, College Station, TX). The ‘svyset’ command were used and weight estimation for the complex survey design based on the probability of sampling, the non-response and post-stratification adjustment by ethnicity, age, and gender [21].

### Comparison of regression models

To explore the data, six regression models were used in this study. Initially, data was explored using ordinary least squares (OLS) as a common regression for healthcare utilisation analysis. The count data models considered in this study were Poisson regression, negative binomial (NB) regression, zero-inflated models (ZIM) such as zero-inflated Poisson (ZIP) and marginalized zero-inflated negative binomial (MZINB), and the hurdle or two-part model (probit and truncated at zero negative binomial).

OLS is a common and basic form of regression with a distinct assumption of normality. The most common evidence published using this National Health Morbidity dataset uses OLS for the analysis of continuous outcome variables [21]. However, with the healthcare utilisation concept, Poisson regression, a basic count model, is used. Poisson assumes equi-dispersion of mean and variance [1], while the NB model is equipped for a parameter to account for overdispersion. It is deemed to be a better model for an overdispersion variance to mean. Both, ZIM and hurdle model allow zero and positive counts, but cater to different decision-making processes [22]. In this dataset, the users of the outpatient healthcare service were based on a few assumptions specified by each model. For ZIM, we assumed that all patients had access to outpatient services and affordability was not an issue to obtain care in the Malaysian healthcare setting [23]. Thus, the occurrence of zero in this dataset was assumed to be a true zero, because a person is a non-user as he or she did not get sick within the study duration (sampling zeros). However, frequency zero represents a person who is sick but chooses not to use outpatient healthcare services (structural zeros). For the hurdle model, we assumed that in this dataset, the first visit was on account of the patient, while the subsequent visit was determined by a joint decision of the patient and their healthcare provider [24]. This principal-agent model allows two different processes. While for marginalized-ZINB, allows for differentiation of latent class of zero for ‘not at risk’ individual and ‘at risk’ individual for outpatient utilisation [5].

Model selection was based on a few steps. The initial step was to check for data distribution using Stata command ‘summarize’, ‘detail’. The occurrence of overdispersion may suggest using NB, ZIM, or hurdle models [24]. Akaike information criteria (AIC) and Bayesian information criterion (BIC) used to compare between models [25][22], where lower AIC and BIC values is preferred [26]. An additional step was also taken by conducting the Vuong test, where a positive value indicates that zero-inflation is appropriate for the said model rather than using a single-equation count model [27] (i.e., Poisson vs. ZIP, NB vs. ZINB). Corrected Vuong test accounting for AIC and BIC value was conducted using an updated “*zipcv”*and zinbcv, [27] and “mzinb” Stata command [28] Root Mean Square Error (RMSE) calculated together and presented with R2 for goodness-of-fit measures [29]. A comparison of the observed and predicted values was also compared [29].

## Results

The frequency distribution of outpatient visits showed that 90% of the population were non-users, while 8.58% utilized healthcare services only once, followed by a smaller percentage of other counts as in Table 1. The maximum distribution of outpatient visits was 25 visits (0.01%) over 14 days. Overdispersion of the mean and variance was observed. Figure A provided as supplementary file showed the skewness of 14.9569 and kurtosis = 363.3711, a large value indicating a positive skew distribution and a high-peak of data distribution [30] .

**Table 1:** Frequency distribution of outpatient visits (number of observations = 11,674)

Total number of outpatient visit (n = 11,674)	Frequency	Percent (%)
0	10,467	89.66
1	1,002	8.58
2	121	1.04
More than 3	84	0.72
ỵ(mean)	0.14	
s^2 y (variance)	0.39	

Table 2 shows the variables in each category of the Andersen model. The initial model had 14 independent variables. For predisposing factors: states (13 States and 3 Federal territory) [31], age, ethnic group (5 major ethnic group) [32], sex and education. The six enabler factors comprise of working status, percentage (50%) of working adults in household, government coverage, employer medical coverage, household income quintile and total household monthly income (ln). Need factors were self-reported health problems, perceived health status, and number of non-communicable diseases (NCDs) diagnosed by health care workers. For final model, manual variable selection was performed and reiteration of regrouping some variables in case if the model can be improved also conducted. The instrumental variables were used consistently across all models.

**Table 2:** Summary statistics of the variables used in the demand equation

Variable	Frequency(%)	Mean(± SD)
***Predisposing factors***		
State		
Johor	1052 (9.01)	
Kedah	669 (5.73)	
Kelantan	709 (6.07)	
Melaka	636 (5.45)	
Negeri Sembilan	653 (5.59)	
Pahang	745 (6.38)	
Penang	688 (5.89)	
Perak	578 (4.95)	
Perlis	667 (5.71)	
Selangor	1324 (11.34)	
Terengganu	730 (6.25)	
Sabah	855 (7.32)	
Sarawak	710 (6.08)	
Federal Territory of Kuala Lumpur	563 (4.82)	
Federal Territory of Labuan	643 (5.51)	
Federal Territory of Putrajaya	452 (3.87)	
Age (years)		44.83 (± 16.55)
Ethnic group		
Malay	7,613 (65.21)	
Chinese	1,483 (12.7)	
Indian	753 (6.45)	
Bumiputera Sabah	651 (5.58)	
Bumiputera Sarawak	488 (4.18)	
Others ethnic	686 (5.88)	
Sex		
Male	5, 517 (47.26)	
Female	6,157 (52.74)	
Education level		
No formal education	679 (5.82)	
Primary education	2,540 (21.76)	
Secondary education	5,593 (47.91)	
Tertiary education	2,862 (24.52)	
Marital status		
Not married*	3,744 (32.07)	
Married	7,930 (67.93)	
***Enabling factors***		
Working Status		
No	4,857 (41.61)	
Yes	6,817 (58.39)	
Percentage (50%) of working adults in Household		
No	8,130 (69.64)	
Yes	3,544 (30.36)	
Government Coverage		
No	8,760 (75.04)	
Yes	2,914 (24.96)	
Employer Coverage		
No	9,506 (81.43)	
Yes	2,168 (18.57)	
Household income quintile		
Poorest quintile	2,500 (21.42)	
Second quintile	2,291 (19.62)	
Third quintile	2,335 (20. 0)	
Fourth quintile	2,301 (19.71)	
Richest quintile	2,247 (19.25)	
Total household income (ln)		7.52(± 1.72)
***Health need factors***		
Had any self-reported health problem		
No	8,130 (69.64)	
Yes	3,544 (30.36)	
Perceived health status		
Excellent & good	8, 751 (74.96)	
Fair	2,639 (22.61)	
Poor & Very poor	284 (2.43)	
Number of diagnosed NCD**		
0	8,363 (71.64)	
1	1,517 (12.99)	
2	1,036 (8.87)	
3	758 (6.49)	

*Not married includes single/widower/divorcee

**NCD: Non-communicable disease, any combination of diabetes, hypertension and hypercholesterolemia

**Fig. 1:**
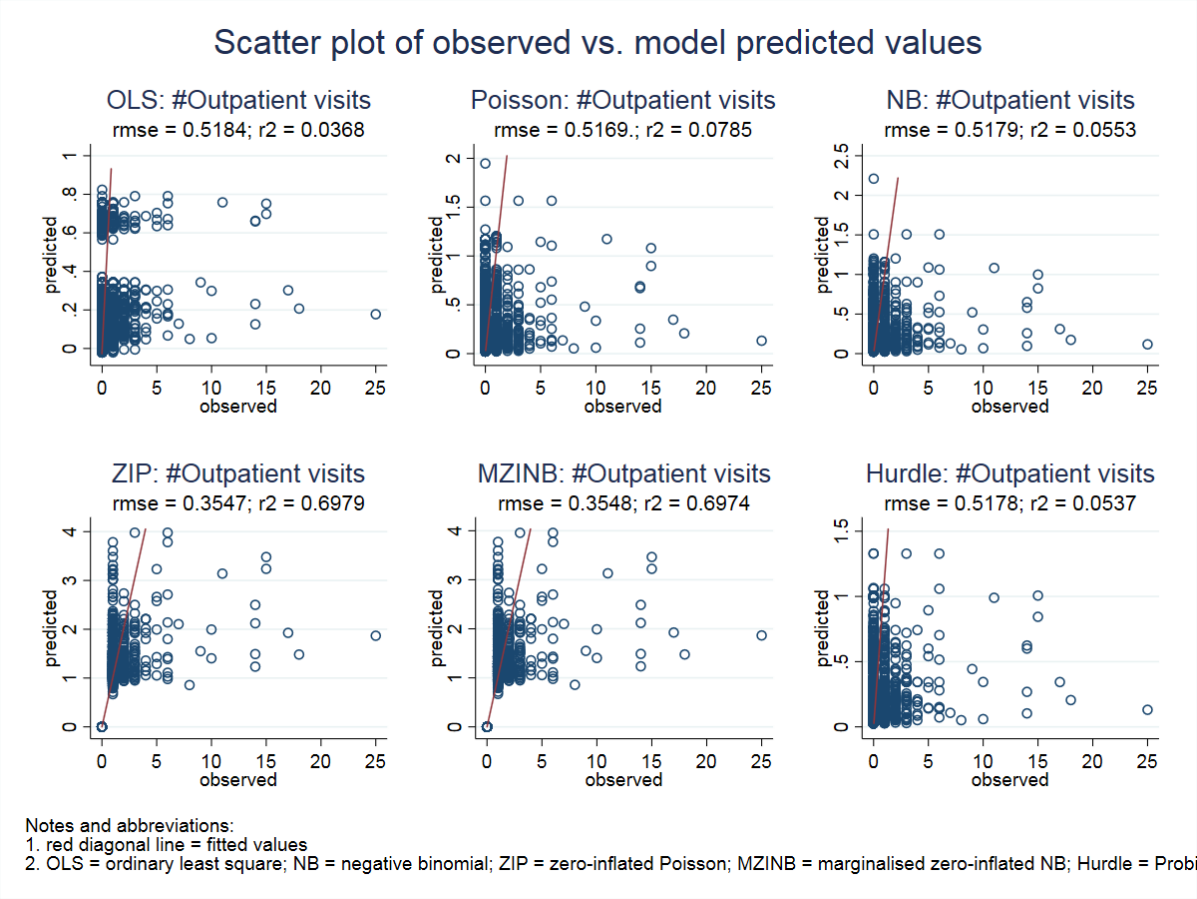
Scatter plot of observed vs. predicted values

To select the best model, we used model selection criteria based on the AIC and the BIC (Table 3). This table also indicates ZIM both ZIP and MZINB show the smallest log likelihood (LL), AIC, and BIC, indicating that the zero-inflated models were preferred. The positive Vuong test confirms that the ZIM is superior to its respective single-equation count model. In this case, the Vuong test with the AIC and BIC correction for both ZIM (ZIP and MZINB) yielded positive values (*p* < 0.001), which corresponds to a statistically significant selection of the zero-inflated model. Measures of goodness-of-fit using RMSE showed ZIM: ZIP (0.3547) and MZINB (0.3548) had a smallest value and R2 of 0.69. Smaller RMSE, reflect the smaller bias between predicted and observed values for each count on the count models considered as depicted in Fig. 1. It shows the distribution of the observed count and predicted count in the models respectively. The red diagonal line served as the best-fitted line for the counts. Additional Figure B: Scatterplot of predicted values vs. residuals and Figure C: QQ-plot of the residuals provided as Supplementary files.

In Fig. 1,

**Table 3:** **Comparisons across all models using LL, AIC and BIC**.

Test statistic	Model					
	**OLS**	**Poisson**	**Negative Binomial (NB)**	**ZIP**	MZINB*	**Hurdle (Probit & NB)**
LLa	-11,743	-5,186	-4,608	-1,698	-1,680	-5,624
AICa	23,541	10,425	9,272	3,455	3,420	11,355
BICa	23,740	10,624	9,478	3,668	3,641	11,753
RMSEa	0.5184	0.5169	0.5179	0.3547	0.3548	0.5178
R2a	0.0368	0.0785	0.0553	0.6979	0.6974	0.0537
Vuong testb						
Uncorrected	-	-	-	1259.9c	1984.8c	-
AIC	-	-	-	1259.9c	1984.8c	-
BIC	-	-	-	1259.9c	1984.8c	-

Notes : Abbreviation: LL = log likelihood; AIC = Akaike’s information criterion; BIC = Bayesian information criterion; RMSE = root mean square error, R2 = r-square

^a^ Lower LL, AIC, and BIC were preferred. Lower RMSE and higher R2 values indicate lesser prediction errors

^b^ Positive Vuong statistics value indicates zero-inflated model is more appropriate than conventional

^c^ Statistical significance at p < 0.001

* indicates preferred model

Thus, we are confident in choosing ZIM of either ZIP or MZINB as the appropriate model based on the lowest AIC and BIC values with a positive Vuong test (*p* < 0.001), Table 4 lists the coefficients of one ZIM best-fit model, MZINB. Through regression modeling, the final model included four instrumental variables. States, ethnicity, household income quintile, and perceived health status were significantly associated with the total number of outpatient visits. This study found that the major ethnic groups: Malay, Chinese and India, and those with perceived health status of “poor and very poor,” had significantly higher number of total outpatient visits. Adult population in certain states in Peninsular Malaysia; Johor, Melaka, Pahang and Terengganu showed a significant outpatient utilisation parallel with significant outpatient utilisation among Sabah and Sarawak population.

**Table 4:** Estimated coefficients for the best fit model, MZINB

Variable	Coef.	SE	*p*-value	95% CI	
				**Lower**	**Upper**
***Predisposing factors***					
State					
Johor	0.326	0.135	0.016	0.061	0.591
Kedah	0.083	0.153	0.589	-0.217	0.382
Kelantan	0.193	0.160	0.228	-0.121	0.507
Melaka	0.411	0.172	0.017	0.075	0.747
Negeri Sembilan	0.264	0.141	0.061	-0.012	0.540
Pahang	0.295	0.144	0.041	0.012	0.577
Penang	*Ref*				
Perak	0.307	0.143	0.031	0.028	0.587
Perlis	0.132	0.247	0.593	-0.352	0.617
Selangor	0.171	0.123	0.165	-0.070	0.413
Terengganu	0.709	0.143	< 0.001	0.429	0.988
Sabah	0.669	0.144	< 0.001	0.387	0.951
Sarawak	0.551	0.144	< 0.001	0.269	0.834
Federal Territory of Kuala Lumpur	0.227	0.161	0.157	-0.088	0.542
Federal Territory of Labuan	0.340	0.363	0.349	-0.371	1.050
Federal Territory of Putrajaya	0.326	0.296	0.271	-0.255	0.906
Ethnic group					
Malay	0.410	0.119	0.001	0.176	0.643
Chinese	0.401	0.117	0.001	0.173	0.630
Indian	0.411	0.140	0.003	0.137	0.686
Bumiputera Sabah	-0.073	0.163	0.654	-0.393	0.247
Bumiputera Sarawak	*Ref*				
Others ethnic	0.314	0.147	0.032	0.026	0.601
***Enabling factors***					
Poorest quintile	*Ref*				
Second quintile	0.101	0.066	0.123	-0.027	0.230
Third quintile	0.068	0.065	0.296	-0.059	0.195
Fourth quintile	0.186	0.069	0.007	0.052	0.320
Richest quintile	0.234	0.067	< 0.001	0.103	0.366
***Health need factors***					
Had any self-reported health problem					
Perceived health status	*Ref*				
Excellent & good	0.032	0.045	0.487	-0.057	0.120
Fair	0.586	0.069	< 0.001	0.451	0.722
Poor & Very poor	-0.563	0.174	0.001	-0.905	-0.221
**Intercept**	0.326	0.135	0.016	0.061	0.591

## Discussion

Outpatient utilisation data from the NHMS 2019 survey have large zeros, non-negative integers, and continuous data with discrete events. In this dataset, zeros accounted for 89.96% of the total. Approximately 8.58% of the respondents made one outpatient visit, while 1.69% of the population made follow-up outpatient visits. Our data showed a large excess of zeros with a long positive skewed tail, with a maximum of nine outpatient visits over 14 days. This is consistent with other survey data (24, 25) in Asia. Neighboring countries in Indonesia have large zeros amounting to 85% outpatient visits in public facilities and 92% in private facilities [33] in a four-week reference period. Similarly, the Jordan National Health Survey captured 80% of zero [34] with a fourteen-day recall period for outpatient care. In contrast, a study in Norway with a 12 month reference period recorded 78.5 − 86.2% of outpatient utilisation [35]. These examples show that a shorter reference period results in a larger zero. A large zero with a short reference period is inevitable because of the survey design. This study included a 14-day reference period. This study makes assumptions about the two types of zeros— frequency zeros due to no outpatient visits throughout the reference period and a true zero that might be due to no illness or presence of illness but not seeking outpatient care.

The predictors of one outpatient visit are usually determined using OLS. However, count variables, especially those involving healthcare data, rarely meet the distributional assumptions of ordinary least square regressions [24] of normality and constant variance. The OLS results depicted the highest LL, AIC, and BIC in this study. This can result in inaccurate estimates of standard errors, p-values, and confidence intervals. However, a recently published local study [21] of oral healthcare has taken measures to limit the data analysis for one visit and make the assumption that zero occurrences are true zero.

In our study, Poisson’s stand as a basic count model has a strict condition of equal variance and mean [36] and an outcome variable with a Poisson distribution. Owing to its distinct characteristics, Poisson is unsuitable for this data distribution. Our outcome variable has an excess of zero in front, with a long positive tail. While NB caters for overdispersion of mean and variance [37] [38], comparing the value of LL between NB and Poisson shows a smaller LL value in NB than in Poisson. These information criteria were used to help determine appropriate models. The lower the AIC and BIC values, the better the model.

Because NB is unable to cater to overdispersion due to excessive zero, ZIM is considered as an alternative modeling strategy. In our data, ZIM showed better LL, AIC, and BIC values than the hurdle model. Across all models, ZIM was deemed suitable as evidence by the smallest values of LL, AIC, and BIC. The results of goodness-of-fit with smaller RMSE value and better R2 value also preferred ZIM. Our findings are also consistent with other findings that used ZIM [39][40]. The different underlying theories and processes of ZIM and hurdle models also serve as a basis for model selection between these two models. In our health system setting, follow-up visits are usually scheduled by healthcare professionals, especially in a public healthcare setting. Thus, it is more appropriate to use the ZIM.

The Vuong test was used to determine whether estimating a zero-inflation component is appropriate or whether a single equation count model should be used [27]. The result of Stata using Vuong is biased toward supporting the zero-inflation model. The results of both corrected and uncorrected Vuong tests show a positive value, indicating the selection of ZIP. However, with the implementation of a new *zipcv* and *zinbcv* Stata command, there are no significant differences. This study reported no large differences compared to previous studies [27]. In this study, we utilized traditional ZIP and MZINB in exploring the best-fit model for the said data. MZINB used as to cater for unobservable latent classes pertaining to the count zero[5]. Lower LL, AIC, BIC values, and positive Vuong Test, significant p-value together with smaller RMSE value and R2 yielded almost a similar value for both ZIM for our data.

In this study, states, ethnicity, household income quintile and perceived health statuswere significant ddeterminants of closely related healthcare utilisation. This trend is visible in numerous other studies that show that wealth are associated with healthcare utilisation [41]. Socioeconomic factors reflected by household income quintile play an important role in outpatient healthcare utilisation [42, 43]. A population with need factors seeks medical outpatient care. In our study, the perceived health status seems to be a significant factor for seeking outpatient care. This concurs with other studies, as perceived health status increases healthcare utilisation especially in an outpatient setting[44].

The strength of this study lies in its utilisation of national healthcare data. It reflects the entire Malaysian population regardless of citizenship. The NHMS is conducted every four years; thus, it is the best available data reflecting the accuracy and timeliness of healthcare utilisation. The model constructed in this study was adapted to meet the characteristics and population data collected by assimilating the Andersen’s Behavioral Model of Health Services Use. However, this study did not explore the subsequent frequency of outpatient visits using either public or private facilities. In addition, details of the types of government coverage were not specifically explored. This could be an interesting area to explore, given that the government progressively increases initiatives to increase access to outpatient utilisations either in government or private facilities to achieve Universal Health Coverage (UHC).

## Conclusion

Our study demonstrated the statistical advantages of count data model approaches over traditional OLS. The overdispersion shows violations of the underlying assumptions of normality and constant variance when using OLS. In practice, count data models are relatively easy to interpret using Stata. However, we are aware that these techniques are not widely used. Therefore, this study of count data strategies guides and encourages the appropriate use of models in healthcare utilisation studies.

## Electronic supplementary material

Below is the link to the electronic supplementary material.

Supplementary Material 1

Supplementary Material 2

Supplementary Material 3

## Data Availability

The datasets used and/or analysed during the current study are available from the corresponding author on reasonable request upon permission from Director General of Health, Malaysia.
