# Supplementary figures and images for: Count data models for outpatient health services utilisation
Source: BMC Med Res Methodol. 2022 Oct 5;22:261. doi: 10.1186/s12874-022-01733-3 (PMC9533534; doi:10.1186/s12874-022-01733-3)

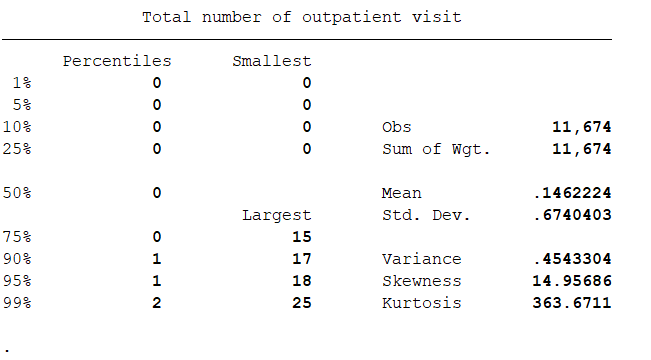

Supplement: Supplementary file 1 — Supplementary Material 1 [file 12874_2022_1733_MOESM1_ESM.png]

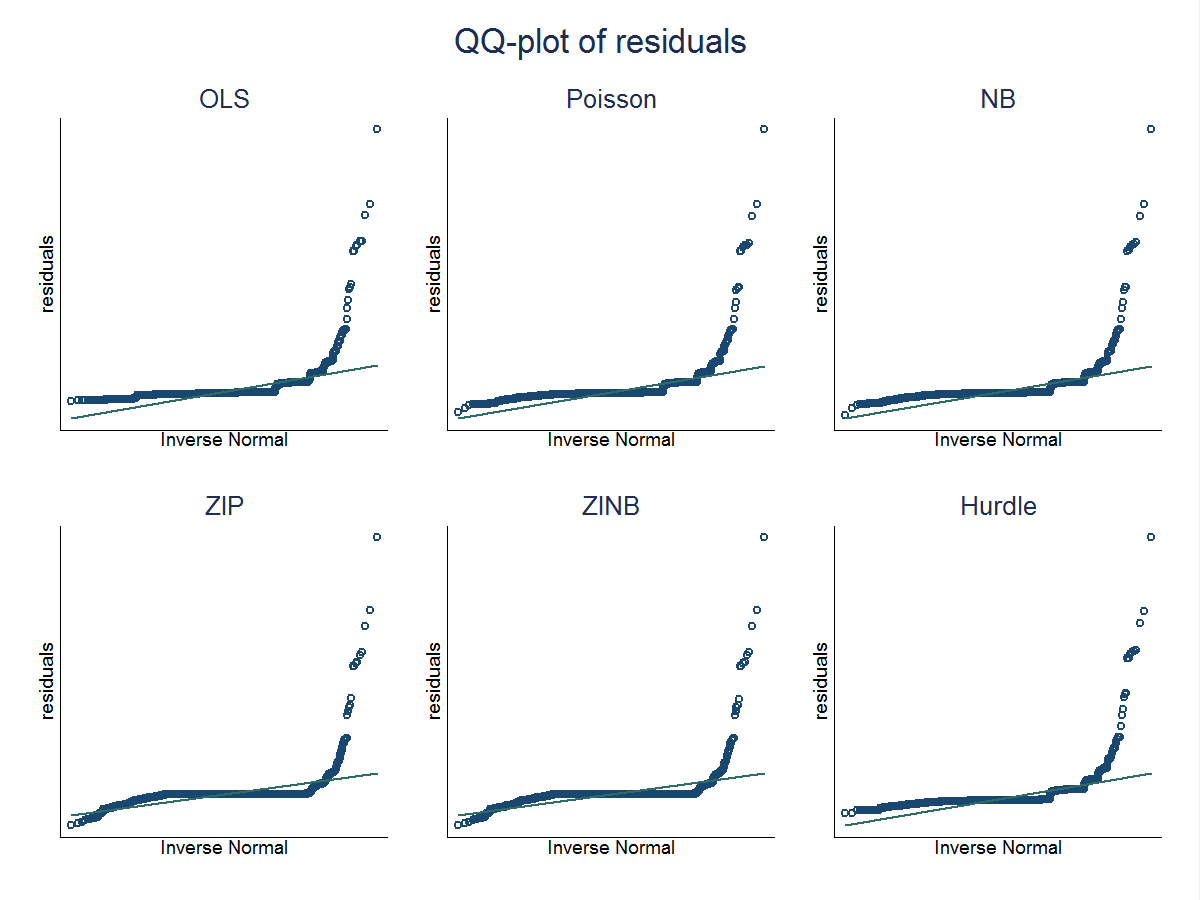

Supplement: Supplementary file 2 — Supplementary Material 2 [file 12874_2022_1733_MOESM2_ESM.png]

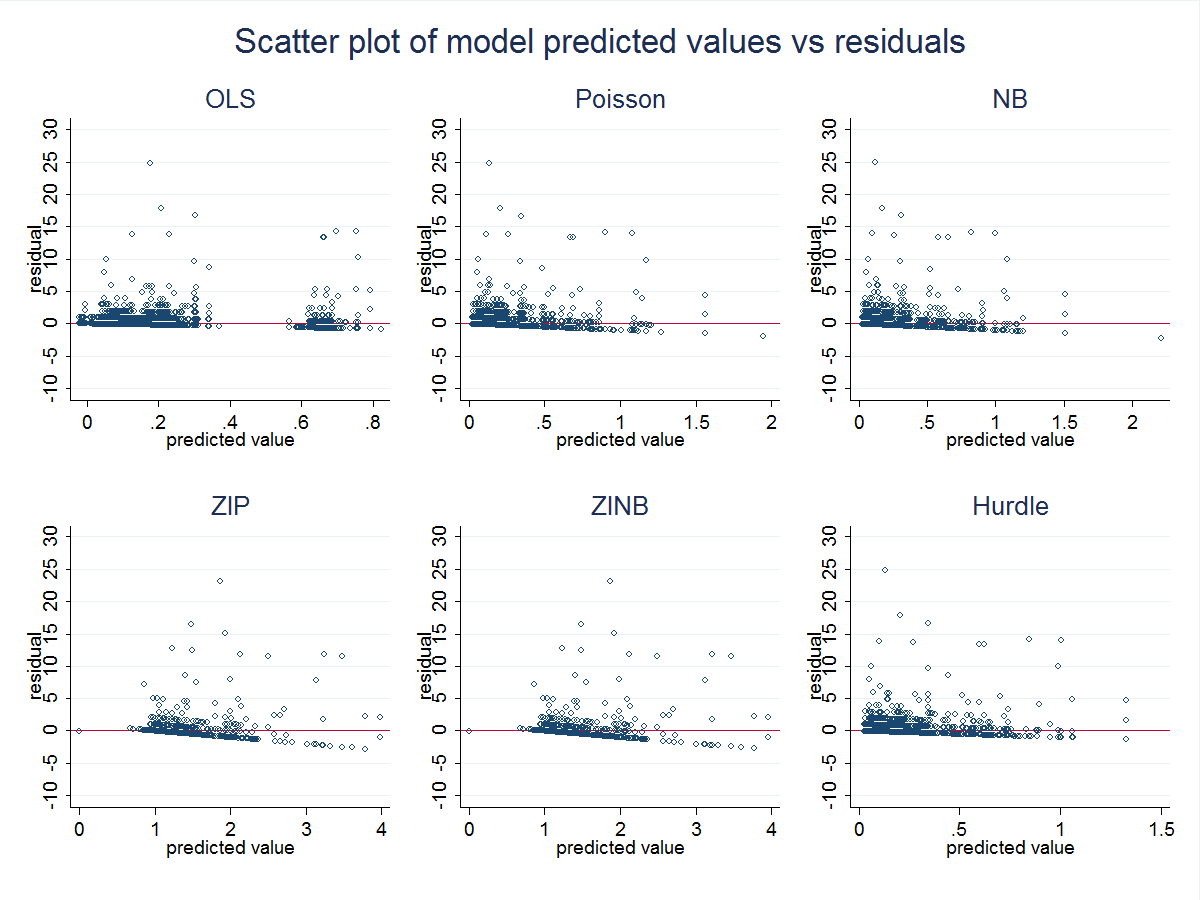

Supplement: Supplementary file 3 — Supplementary Material 3 [file 12874_2022_1733_MOESM3_ESM.png]
